# Timing Matters in Hip Fracture Surgery: Patients Operated within 48 Hours Have Better Outcomes. A Meta-Analysis and Meta-Regression of over 190,000 Patients

**DOI:** 10.1371/journal.pone.0046175

**Published:** 2012-10-03

**Authors:** Lorenzo Moja, Alessandra Piatti, Valentina Pecoraro, Cristian Ricci, Gianni Virgili, Georgia Salanti, Luca Germagnoli, Alessandro Liberati, Giuseppe Banfi

**Affiliations:** 1 Department of Biomedical Sciences for Health, University of Milan, Milan, Italy; 2 University of Milan, Milan, Italy; 3 Clinical Epidemiology Unit, IRCCS Galeazzi Orthopedic Institute, Milan, Italy; 4 Epidemiological Observatory, ASL Milano, Milan, Italy; 5 Department of Oto Specialised Surgical Sciences, University of Florence, Florence, Italy; 6 Clinical and Molecular Epidemiology Unit and Clinical Trials and Evidence-Based Medicine Unit, Department of Hygiene and Epidemiology, School of Medicine, University of Ioannina, Ioannina, Greece; 7 Department of Oncology, Hematology and Respiratory Diseases, University of Modena and Reggio Emilia, Modena, Italy; Johns Hopkins Bloomberg School of Public Health, United States of America

## Abstract

**Background:**

To assess the relationship between surgical delay and mortality in elderly patients with hip fracture. Systematic review and meta-analysis of retrospective and prospective studies published from 1948 to 2011. Medline (from 1948), Embase (from 1974) and CINAHL (from 1982), and the Cochrane Library. Odds ratios (OR) and 95% confidence intervals for each study were extracted and pooled with a random effects model. Heterogeneity, publication bias, Bayesian analysis, and meta-regression analyses were done. Criteria for inclusion were retro- and prospective elderly population studies, patients with operated hip fractures, indication of timing of surgery and survival status.

**Methodology/Principal Findings:**

There were 35 independent studies, with 191,873 participants and 34,448 deaths. The majority considered a cut-off between 24 and 48 hours. Early hip surgery was associated with a lower risk of death (pooled odds ratio (OR) 0.74, 95% confidence interval (CI) 0.67 to 0.81; P<0.000) and pressure sores (0.48, 95% CI 0.38 to 0.60; P<0.000). Meta-analysis of the adjusted prospective studies gave similar results. The Bayesian probability predicted that about 20% of future studies might find that early surgery is not beneficial for decreasing mortality. None of the confounders (e.g. age, sex, data source, baseline risk, cut-off points, study location, quality and year) explained the differences between studies.

**Conclusions/Significance:**

Surgical delay is associated with a significant increase in the risk of death and pressure sores. Conservative timing strategies should be avoided. Orthopaedic surgery services should ensure the majority of patients are operated within one or two days.

## Introduction

Hip fractures are common and serious: they always cause short-term pain, disability and can lead to longer-term pain, disability and even deformity. Mortality rate is estimated to be 5–10% at one month and 12–27% at one year from surgery [1]. Recent data from the UK, USA and Canada show that patients are mostly older than 75 years and female [2], [3], [4]. Mortality in the elderly may reach 10% at one month, 20% at four months and 30% at one year [5]. These patients are the frailest among those who are admitted to hospital, and their outcomes are likely to depend closely on how their care is managed.

In the last decade efforts have been made to boost our knowledge of the prognostic factors influencing the course and management of hip fracture. As the majority are treated surgically, time to surgery may be decisive. Some studies report that pre-operative delay might lead to an increase in mortality and adversely influence other clinical outcomes such as infection and pressure sores [6], [7], [8], [9]. Clinical guidelines recommend immediate reparative surgery, within 24–48 hours from hospital admission [10], [11]. However, several observational studies found no association between time and mortality and concluded that further research is needed on whether functional outcomes are worsened by delaying surgery [12], [13], [14]. This approach is supported by geriatricians who offer an explicit rationale for postponing surgery: delay may be necessary and beneficial for stabilizing patients with co-morbidities [12].

**Figure 1:**
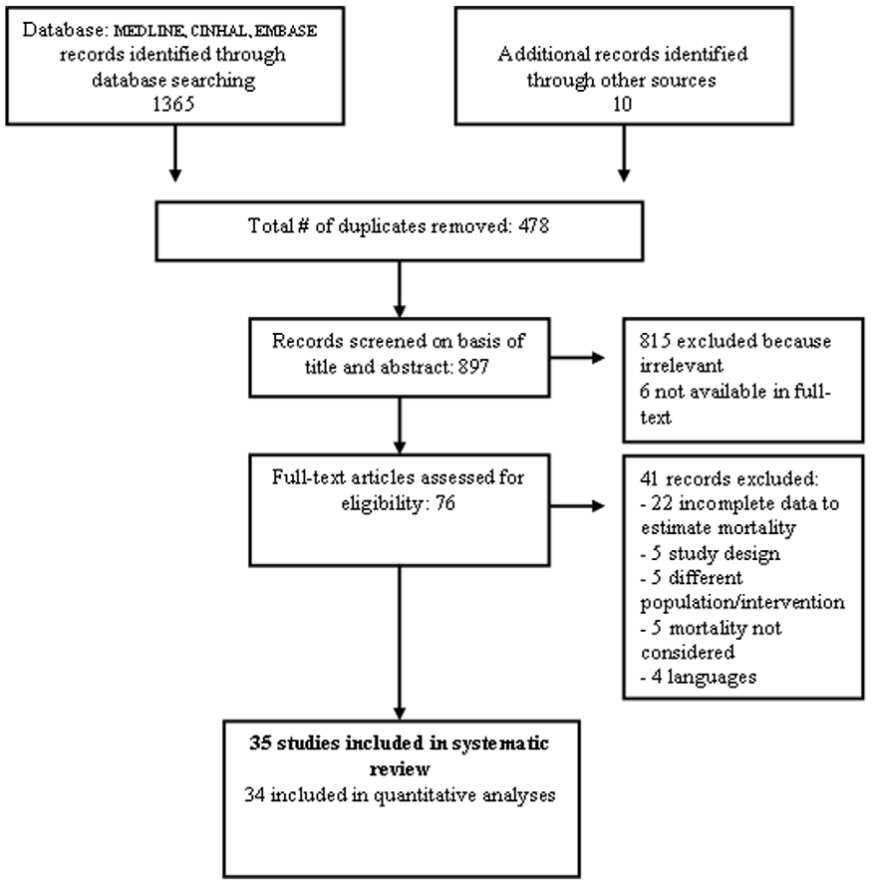
Selection for studies exploring the association between mortality and optimal time to surgery in patients with hip fractures.

**Table 1:** Characteristics of included studies.

Source	Design	Source of data	Data source for pre-operative time and confounding factor	Country	No of participants	Average age	% Female	Optimal Time (hours)	Methodology score
**Al Ani 2008**	Prospective	Two centres	Clinical records	Sweden	744	81	73	24	7
**Bergeron 2006**	Retrospective	One centre	Unclear	Canada	977	81	74	24	7
**Bredahl 1992**	Retrospective	One centre	Clinical records	Denmark	778	79	73	12	5
**Carretta 2011**	Retrospective	One centre	Administrative data	Italy	1320	83	77	48	7
**Davis 1988**	Prospective	Two centres	Clinical records	USA	230	81	82	48	6
**Dorotka 2003**	Prospective	One centre	Clinical records	Austria	182	78	76	24	7
**Doruk 2003**	Prospective	One centre	Clinical records	Turkey	65	76	64	120	7
**Elliott 2003**	Prospective	Two centres	Clinical Records	Ireland	1780	NR	77	24	9
**Franzo 2005**	Retrospective	18 centres	Administrative data	Italy	6629	82	81	48	8
**Gdalevich 2004**	Retrospective	One centre	Clinical records	Israel	651	NR	75	48	8
**Grimes 2002**	Retrospective	20 centres	Clinical records**	USA	8383	80	79	24	8
**Hamlet 1997**	Retrospective	One centre	Clinical records	USA	171	77	80	24	6
**Holt 2008**	Prospective	22 centres	Clinical records	UK	18692	NR	79	24	9
**Kenzora 1986**	Retrospective	One centre	Clinical records	USA	406	76	76	24	6
**Maggi 2009**	Prospective	Nine centres	Clinical records	Italy	2473	82	79	24	8
**Majumdar 2006**	Retrospective	Multicentre*	Administrative data	Canada	3864	82	71	24	8
**Mc Guire 2004**	Retrospective	Multicentre*	Administrative data	USA	18208	82	79	48	8
**Moran 2005**	Prospective	One centre	Clinical records	UK	2148	80	76	96	9
**Mullen 1989**	Prospective	One centre	Clinical records	USA	400	79	NR	24	6
**Novack 2007**	Retrospective	Seven centres	Administrative data	USA	3815	82	73	48	9
**Orosz 2004**	Prospective	Four centres	Clinical records	USA	1576	82	81	24	9
**Parker 1992**	Prospective	One centre	Clinical records	UK	468	81	83	48	7
**Peleg 2011**	Retrospective	Seven centres	Administrative data	Israel	6442	NR	NR	48	6
**Radcliff 2008**	Retrospective	181 centres	Administrative data	USA	5682	77	0	96	6
**Rademakers 2007**	Retrospective	One centre	Clinical records	Netherlands	722	82	76	12	5
**Rae 2007**	Prospective	One centre	Clinical records	Australia	222	79	72	48	8
**Roos 1996**	Retrospective	Multicentre*	Administrative data	USA	26213	81	79	48	5
**Sexson 1988**	Retrospective	One centre	Clinical records	USA	300	NR	77	24	8
**Smektala 2008**	Prospective	268 centres	Clinical records	German	2916	NR	80	48	8
**Siegmeth 2005**	Prospective	One centre	Clinical records	UK	3628	81	NR	48	8
**Stoddart 2002**	Retrospective	One centre	Clinical records	New Zealand	138	83	75	24	7
**Sund 2005**	Retrospective	Multicentre	Administrative data	Finland	16881	82	75	48	9
**Swanson 1998**	RCT	One centre	Clinical records	USA	71	79	78	48	NA
**Verbeek 2007**	Retrospective	One centre	Clinical records	Netherlands	192	80	77	24	9
**Weller 2005**	Retrospective	One centre	Administrative data	Canada	57315	78	75	24	8

* Number of participating centres not reported. **Data already collected for other purpose. NR Not Reported. NA Not applicable.

A large number of observational studies have explored prognostic factors influencing hip fracture surgery, but with discordant results. Reasons might depend on the variability in adjusting for different confounders among studies, the lack of a consistent choice of the reference group and use of a common surgical delay cut-off.

**Table 2:** Methodological Quality Assessment of Observational Studies Based on the Newcastle-Ottawa (NOS) scale.

Study ID	Selection		Comparability		Outcome		Total score*
	Representativeness of early cohort	Selection of delay cohort	Controlled for age	Controlled for co-morbidities	Follow- up length	Adequacy follow-up	
**Al Ani 2008**	*	*			*	*	7
**Bergeon 2006**	*	*	*	*			7
**Bredahl 1992**					*	*	5
**Carretta 2011**		*	*		*	*	7
**Davis 1988**	*	*			*		6
**Dorotka 2003**	*	*			*	*	7
**Doruk 2003**	*	*			*	*	7
**Elliott 2003**	*	*	*	*	*	*	9
**Franzo 2005**	*	*	*	*	*		8
**Gdalevich 2004**	*	*	*	*	*		8
**Grimes 2002**	*	*	*	*	*		8
**Kenzora 1986**	*	*			*		6
**Hamlet 1997**	*	*			*		6
**Holt 2008**	*	*	*	*	*	*	9
**Maggi 2009**	*	*	*	*	*		8
**Majumdar 2006**	*	*	*	*	*		8
**Mc Guire 2004**	*	*	*	*		*	8
**Moran 2005**	*	*	*	*	*	*	9
**Mullen 1989**	*	*			*	*	6
**Novack 2007**	*	*	*	*	*	*	9
**Orosz 2004**	*	*	*	*	*	*	9
**Parker 1992**	*	*			*	*	7
**Peleg 2011**			*		*	*	6
**Radcliff 2008**			*	*		*	6
**Rademakers 2007**	*	*					5
**Rae 2007**	*	*	*	*		*	8
**Roos 1996**			*			*	5
**Sexson 1988**	*	*		*	*	*	8
**Siegmeth 2005**	*	*		*	*	*	8
**Smektala 2011**	*	*	*		*	*	8
**Stoddart 2002**	*	*			*	*	7
**Sund 2005**	*	*	*	*	*	*	9
**Verbeek 2007**	*	*	*	*	*	*	9
**Weller 2005**	*	*	*	*	*		8

* Total score: sum of row totals plus 3 points scored positively across all studies (see methods section for details).

A meta-analysis published in 2010 investigating the effect of surgical delay on mortality at different follow -up times, found significantly higher all-cause mortality for patients treated surgically more than 24, 48 and 72 hours from admission [15]. We moved from this high-quality meta-analysis to conduct a comprehensive meta-analysis which considered all prospective and retrospective evidence. The aims of this systematic review were: (i) to identify and describe all the studies which assessed whether surgical delay increases the mortality of elderly patients treated for hip fracture; (ii) to see whether delay is associated with increased mortality; (iii) to examine the influence of a wide range of *a priori* selected variables (age, sex, co-morbidities, etc.) using meta-regression; and (iv) to test the robustness of results using a Bayesian approach.

**Figure 2:**
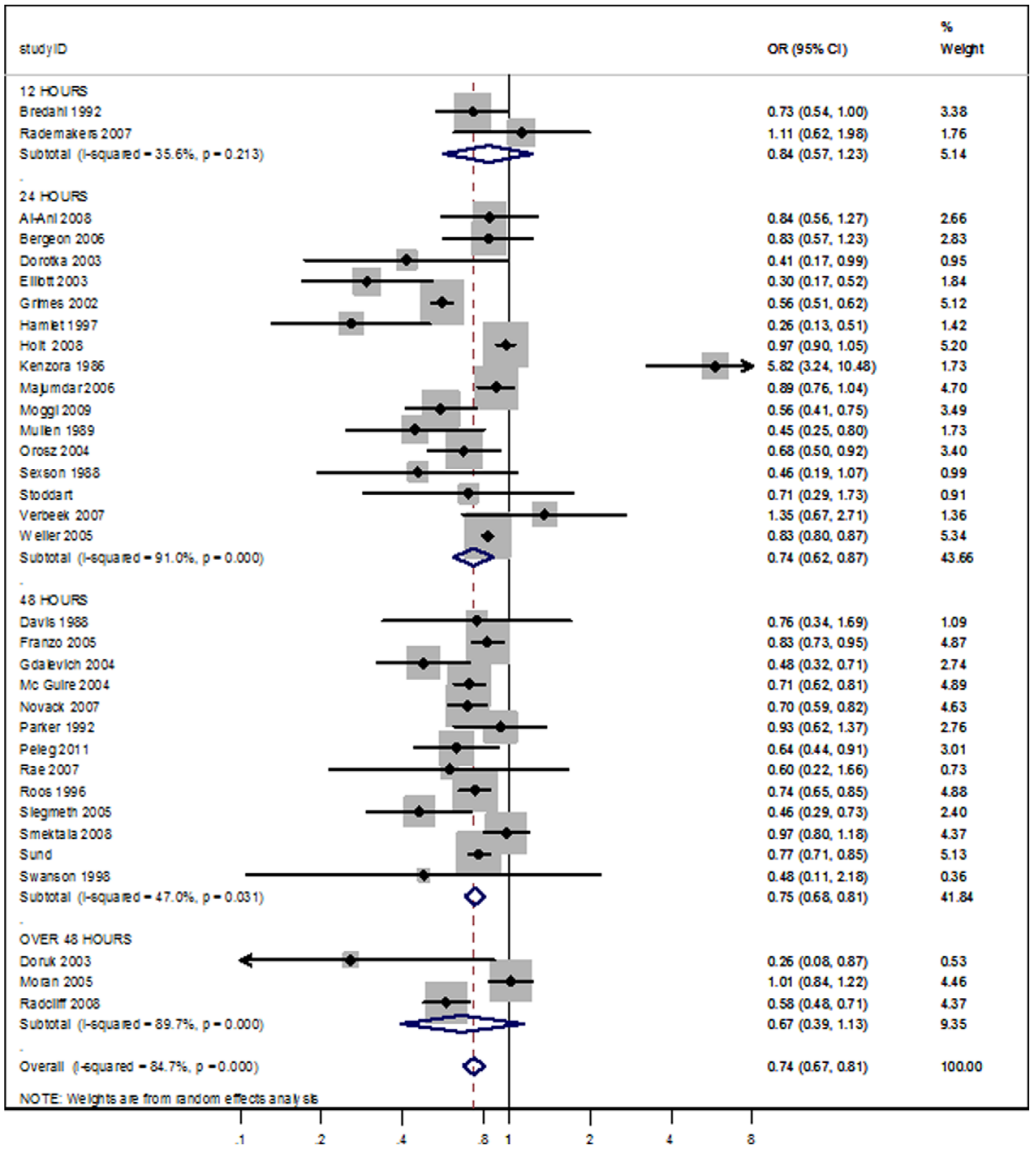
Meta-analysis of Early versus Delayed surgery time according to cut-off points (12, 24, 48, and over 48 hours). Outcome: overall mortality.

## Methods

### Eligibility

Studies were included if they met the following criteria: i) randomized, quasi-randomized (e.g. allocation based on date of admission), prospective and retrospective cohort and case-controlled studies; ii) patients with operated hip fractures; iii) patients aged 65 years or older (median or mean age per study); iv) reporting of timing of hip surgery; v) survival status adequately reported for meta-analysis; vi) published in English, French, Italian or Spanish after 1980. Evidence from controlled observational studies was included: it is unlikely that patients were randomized to receive immediate or postponed surgery to obtain evidence of the mortality-delay association because of ethical concerns. We did not define the optimal surgical delay from hospital admission to reparative surgery *a priori* but accepted what the authors claimed at face value. When authors did not give a cut-off, we arbitrarily selected 24 hours as optimal. Different time cut-offs were used as strata in our analyses.

**Figure 3:**
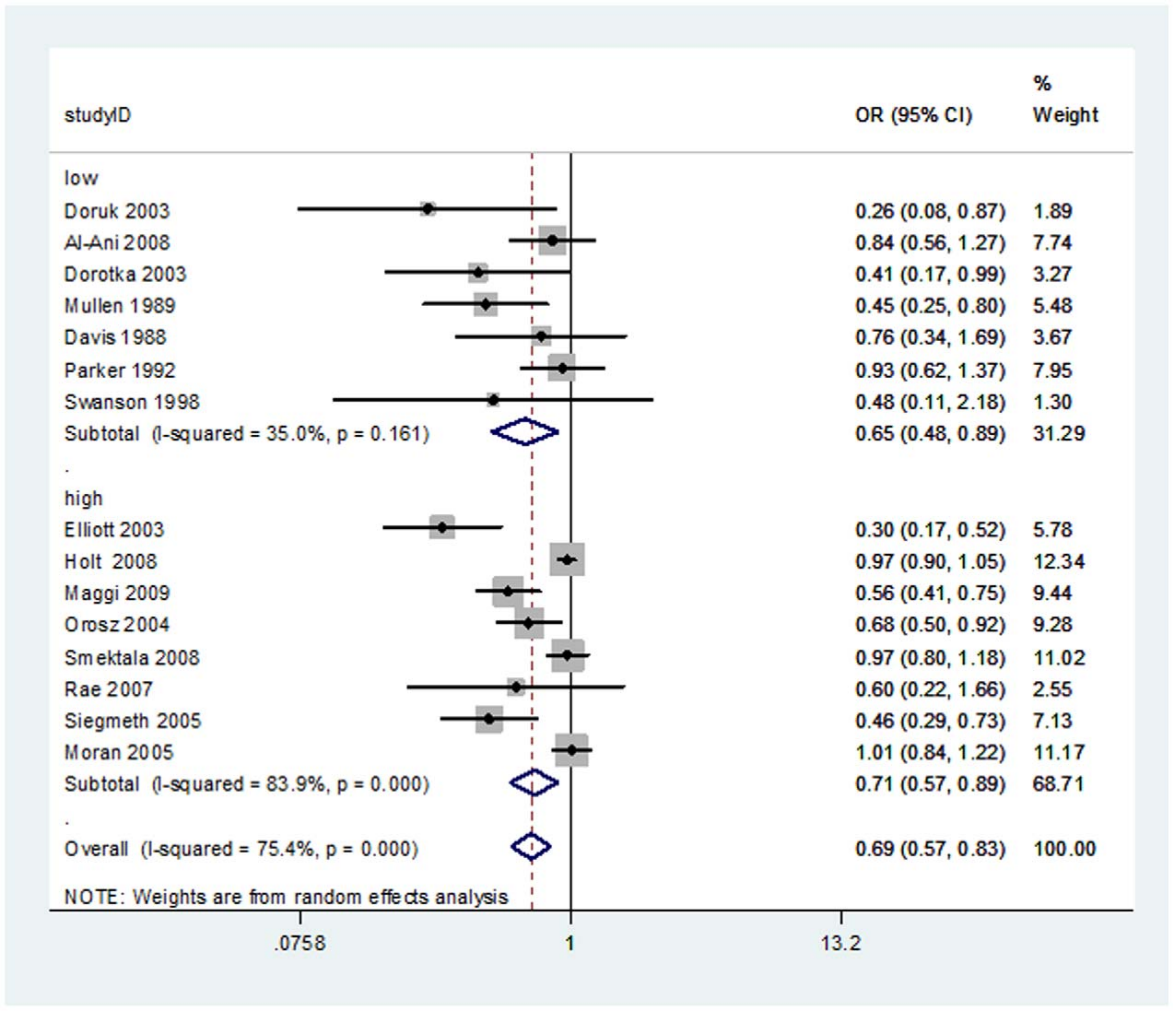
Meta-analysis of high and low-quality adjusted prospective studies comparing early versus delayed surgery time. Outcome: overall mortality.

### Search strategy

Studies were identified by searching electronic databases and scanning reference lists of articles. This search was applied to Medline (1948 – /September 2011), and adapted for Embase (1974 – September 2011) and CINAHL (1982 – December 2011). The strategy was developed using the following key items: hip fracture, arthroplasty, and timing surgery (see Search strategy – *Table* *S1*) [16], [17]. Manual searches of the reference lists of included studies, reviews, meta-analyses and guidelines on hip fracture surgery and prognostic factors were also done.

**Figure 4:**
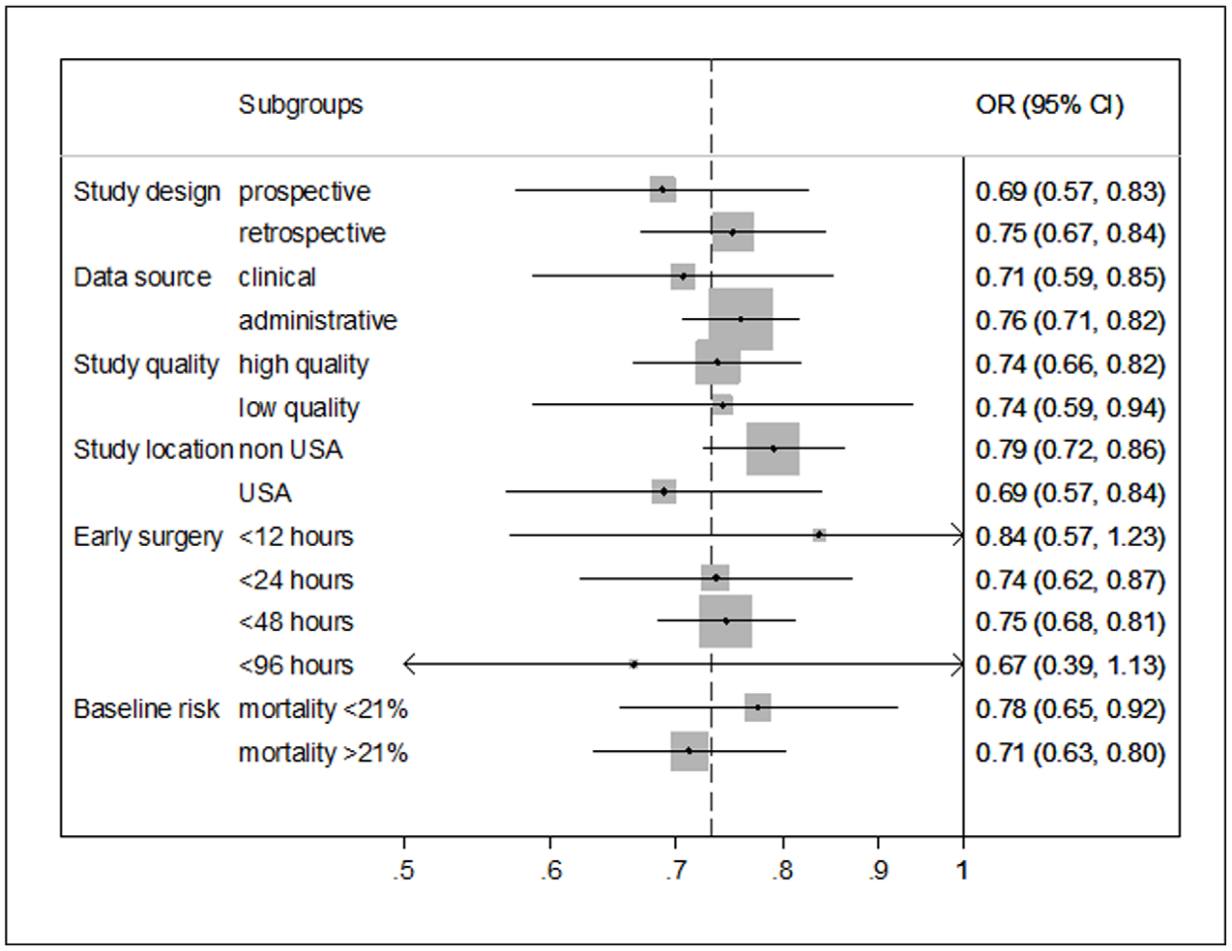
Subgroups analyses of Early and Delayed surgery time for overall mortality.

### Study selection

The literature search was conducted independently and two reviewers (AP and LG) independently searched the literature who then selected potentially eligible studies for inclusion. Disagreements between them were resolved by consensus; if no agreement could be reached, a third author (LM) was called in to decide. The fulltext of all eligible citations was examined in more detail.

### Data Extraction

We developed a data extraction sheet, pilot-tested it on five randomly-selected studies, and refined it accordingly. One review author (AP) extracted the following data from studies included and entered in the data extraction form: study design, study year, participants (age, sex, case-mix and co-morbidities), country of origin and setting. A second author (CR) checked the extracted data to ensure quality. Disagreements were solved by discussion between the two review authors; if no agreement was reached, a third author (LM) could decide.

**Table 3:** Random effects meta-regression analyses of Early and Delayed surgery time for overall mortality.

Characteristic	N. studies	N. participants	OR (95% CI)	p-value
**Continuous variables**				
Age (per 10 years)	28	159772	0.97 (0.36 to 2.63)	0.96
Female prevalence (per 10% more)	32	187885	1.03 (0.92 to 1.16)	0.55
Study year (per 10 years)	34	191873	0.90 (0.7 to 1.18)	0.46
Continuous time (per 24 hours)	34	191873	0.22 (0.00077 to 63.62)	0.59
**Categorical variables**				
*Study design*				
Prospective (reference)	15	35112	1	
Retrospective	20	156761	1.17 (0.82 to 1.66)	0.36
*Data source category*				
Clinical data (reference)	24	46843	1	
Administrative data	10	144053	1.05 (0.72 to 1.53)	0.78
*Study quality*				
8–9 stars (reference)	18	149228	1	
1–7 stars	17	42645	1.05 (0.73 to 1.5)	0.79
*Study location*				
Non-US study (reference)	23	129179	1	
US study	12	62694	0.94 (0.65 to 1.35)	0.74
*Early surgery time cut-off definition*				
<12 hours	2	1500	1.2 (0.55 to 2.65)	0.62
<24 hours (reference)	16	97100	1	
<48 hours	14	85378	0.97 (0.65 to 1.43)	0.87
<96–120 hours	3	7895	0.90 (0.46 to 1.78)	0.77
*Baseline risk*				
Risk<21%	17	85826	1	
Risk>21%	18	106047	0.86 (0.61 to 1.22)	0.40

The primary outcome was unambiguous overall mortality. If applicable short- (<30 days) and long-term mortality (>30 days) were combined. Secondary outcomes were post-operative complications, i.e. infections, pressure sores, post-operative chronic pain, hospital length of stay, and readmission. If necessary, percentages of mortality or other outcomes were converted into frequencies.

**Figure 5:**
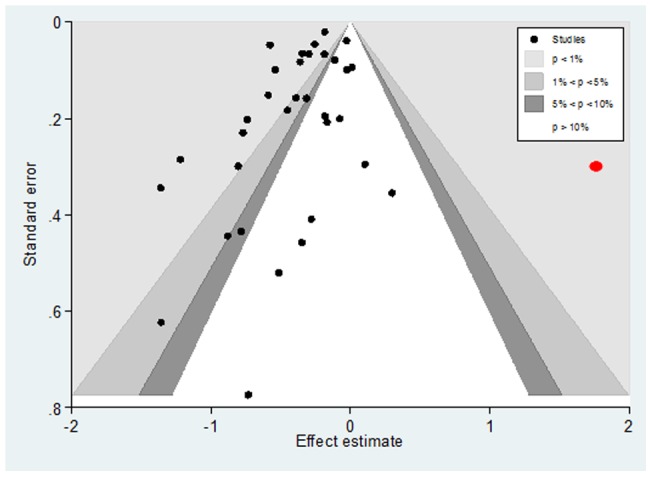
Contour enhanced funnel plot of studies comparing Early and Delayed surgery time for overall mortality. Caption: Kenzora 1986 (in red), while laying in the area of statistical significance favouring late surgery, may have interfered with the effect of small studies in the funnel plot.

For all studies that addressed mortality, we recorded the unadjusted matched odds ratios (OR) for: i) the comparison of early and delayed surgery; ii) whether any adjustment was made for covariates (e.g. age); iii) the adjusted estimate of the ORs and 95% confidence intervals (CIs). When univariate and multivariate adjusted models were available, both were abstracted. Since some studies reported only adjusted hazard ratios, we converted them to ORs using a formula to compute risk ratio from OR [18]. We used as control mortality risk the median control risk based on our primary meta-analysis.

**Table 4:** Similarities and differences between this systematic review and an independent one by Simunovic et al. 2010 [15].

Systematic review	Simunovic 2010	Moja 2012
**Objectives**	We conducted a systematic review and meta-analysis to determine the effect of early surgery on the risk of death and common postoperative complications among elderly patients with hip fracture.	The aims of this systematic review are: (i) to identify and describe all the studies which assessed whether operative delay increases the mortality of elderly patients treated surgically for hip fracture; (ii) to see whether delay is associated with increased mortality; and (iii) to examine the influence of *a priori* selected variables (age, sex, co-morbidities, etc.).
**Results on mortality**		
Overall	16 studies, 13 478 patients, RR 0.55, 95% CI 0.40–0.75, p<0.001, I^2^ = 71%.	34 studies, 191 873 patients, OR 0.74, 95% CI 0.67 to 0.81, *P*<0.0001, I^2^ = 85%.
High-quality studies	5 studies, 4 208 patients, RR 0.81, 95% CI 0.68–0.96, p = 0.01, I^2^ = 0%.	8 studies, 33435 patients OR: 0.81; 95%CI: 0.71 to 0.59, p = 0.0001, I^2^ = 86%.
**Authors’ conclusions on mortality**	Earlier surgery was associated with a lower risk of death and lower rates of postoperative pneumonia and pressure sores among elderly patients with hip fracture. These results suggest that reducing delays may reduce mortality and complications.	Surgical delay is associated with a significant increase in the risk of death and pressure sores. Conservative timing strategies should be limited to patients who may benefit most. Orthopaedic surgery services should ensure the majority of patients are operated between one and two days.
**Methods**		
Outcomes	Mortality, pressure sores, pneumonia, deep vein thrombosis, pulmonary embolism	Mortality, pressure sores
Eligibility criteria	i) Patients 60 years of age or older; ii) who underwent surgery for a low-energy hip fracture; iii) evaluation of preoperative surgical delay; iv) consideration of all-cause mortality as an outcome; and v) prospective design.	i) Randomized, quasi-randomized (i.e. allocation based on date of admission), prospective and retrospective cohort and case-controlled studies; ii) inclusion of patients with operated hip fractures; iii) inclusion of timing of hip surgery; iv) inclusion of patients older than 65 years (median or mean age per study); v) survival status adequately reported for meta-analysis.
Study identification	No language and year restrictions.	Studies published in English, French, Italian or Spanish after 1980.
Risk of bias	Newcastle-Ottawa Scale criteria	Newcastle-Ottawa Scale criteria
Summary statistics	Relative Risk	Odds Ratio
Statistical approaches	Random-effects model of DerSimonian and Laird.	Primary: Random-effects model of DerSimonian and Laird, prediction interval. Secondary: fixed-effect model of Mantel-Haenszel and Bayesian.
Stratification for	Time according to follow-up mortality (short, medium and long-term).	Time according to cut-off points for surgery (12, 24, 48, and over 48 hours).
**Strengths**	Extensive study search Postoperative complications.	Meta-regression and Bayesian meta-analysis.

If a study considered both young patients (<65 years or high-velocity injuries) and old patients, the data were extracted separately if possible, and only the elderly group was considered. If sorting was not possible and at least 90% of the patients in a study were classifiable as elderly, the study was included; if not it was excluded.

### Methodological quality

Methodological quality was independently assessed by two review authors (AP and LG). For observational studies the Newcastle-Ottawa (NOS) scale for cohort and case-control studies was used [19], [20]. This scale has three groups of items: selection, exposure/outcome and comparability. A study can be awarded a maximum of one star for each numbered item in ‘patient selection’ (four items) and ‘exposure’ (three items) and a maximum of two stars in the ‘comparability of study groups’ (two items), for a total of nine stars. Since we were interested in mortality of operated patients, we expected three items would be scored positively across all studies, specifically *Ascertainment of exposure (secure surgical record), Demonstration that outcome of interest was not present at start of study, Assessment of outcome (record linkage)*. In fact in our meta-analysis the NOS scale could have ranged between three and nine. For randomized controlled trials (RCTs) we summarized the risk of bias for mortality across the following specific domains within study: sequence generation, allocation concealment, and incomplete outcome data [21]. We decided *a priori* that only prospective observational studies that met eight or nine of the Newcastle-Ottawa Scale criteria were to be considered of high quality, whereas RCTs were considered of high quality if they satisfied two or more.

### Data analysis

We did an overall quantitative synthesis using all ORs on mortality across all studies, with unadjusted data from each study. The results were pooled using the inverse variance method and ordered by study year. The random effects model described by DerSimonian and Laird [22] was used to synthesize data rather than the fixed effect model because it incorporates intra- and inter-study variability. This model was selected a priori as the meta-analysis was expected to include primarily observational studies with inherently more variability than RCTs. A Mantel-Haenszel OR was also computed using fixed effect and compared to the DerSimonian and Laird estimate to investigate any influence of small study effects on the pooled OR, since the DerSimonian and Laird methods tend to attribute greater weight to small studies with increasing heterogeneity [23]. The degree of heterogeneity between trials was assessed by the I-squared (I^2^) statistic, with its 95% CI for each outcome. We accounted for heterogeneity in the results of our primary meta-analysis, using the prediction interval (estimated in a Bayesian setting) for the true effect in a new study, which describes the full distribution of effects in a random-effects meta-analysis [21].

Since meta-analyses of observational studies often give spuriously precise results, we tested the robustness of results with two approaches. First, we calculated the probability of the OR being less than 1 from the predictive Bayesian interval. Second, we used credibility ceilings, a technique which assumes that methodological limitations implicit in included observational studies cannot give us more than a maximum certainty that an effect is in a particular direction and not null or in the other direction [24].

Sensitivity to prior assumptions was checked in the Bayesian estimation of the pooled OR as well as the predictive interval. We assumed different priors for the shape of the random effects distribution: Gamma (0.001, 0.001) on precision; uniform (0, 50) on inter-study variance tau2; uniform(0, 50) on inter-study standard deviation tau; and three functions of mean intra-study variance: uniform shrinkage on tau2, DuMouchel on tau, and half-normal on tau2.

Further sensitivity analysis was done to account for methodological differences between the studies. Data were synthesized for studies of high and low quality, using adjusted study-specific estimates, preferring the ones with the most extensive adjustment [25].

The extent to which study-level variables explained heterogeneity in predicting mortality was explored by fitting random effects meta-regression models to account for the design (prospective or retrospective), nature of data (administrative or clinical), methodological quality (8–9 stars or 1–7), health status or co-morbidities (to identify patients at high risk of mortality), location of study (United States or other), different optimal time cut-points, age, prevalence of females, study year, and optimal time treated as a continuous variable. The effect of mortality baseline risk was investigated in a Bayesian linear regression model.

We checked for potential publication and small study effects by the contour enhanced funnel plot [26], [27] integrating visual inspection of the plot with the test proposed by Harbord [28].

Statistical analyses were done using Stata v.11 [29] and WinBUGS v.1.3 software [30] *P-*values less than 0.05 were considered statistically significant.

## Results

Database searches yielded 1375 references. Hand-searching produced 10 more. Exclusion of duplicates and irrelevant references left 76 descriptive studies. We excluded 41 studies because of: incomplete data (n = 22), study design (n = 5), different population/intervention (n = 5), mortality not considered as an outcome (n = 5), language (n = 4). We included 35 studies fulfilling our inclusion criteria, all except one [31] providing data for our analyses (*Figure* *1*) [6], [7], [13], [31], [32], [33], [34], [35], [36], [37], [38], [39], [40], [41], [42], [43], [44], [45], [46], [47], [48], [49], [50], [51], [52], [53], [54], [55], [56], [57], [58]. This corresponds to 34 cohort studies, 14 using a prospective design and 20 a retrospective one, and one RCT. summarizes the main features of the 35 articles included, which included 191,873 participants. The number of participants in each trial ranged from 65 to 57,315 (*Table* *1*). The mean age was 80 years (range 76–83 years). The mean proportion of women was 74% (0–83%) in the 32 studies reporting sex. Three studies were published in the 80’s, six in the 90’s and 26 (71.8%) after 2000. Twelve were from the USA, four from UK, three each from Canada and Italy, two each from Israel and Netherlands; Australia, Austria, Denmark, Finland, German, Ireland, New Zealand, Turkey and Sweden provided one study each.

Patient data were collected from clinical records in 24 studies, in ten from national or regional administrative databases, and in one the source was unclear. The optimal surgical delay was 12 hours in two studies, 24 hours in 16, 36 hours in one study, 48 in thirteen, and >72 in three. The quality measured through the NOS scale ranged from five to nine points, with a median of seven (*Table* *2*).

The only RCT was at high risk of bias [57]. Swanson randomized a limited number of patients (71) to a multifaceted intervention named ‘early intervention’. Early surgery, early mobilization, and intensive support by health professionals were the main components, although it is difficult to evaluate their relative roles. The groups did not truly differ in terms of time to surgery: 90% of patients in the intervention group and 80% in the control group were operated within 48 hours. The generation of the randomization sequence and allocation concealment were likely to be inappropriate, possibly being affected by the time of hospital admission.

### Time to surgery and mortality

Patients who underwent early surgery had significantly lower odds of death than those whose surgery was delayed (OR 0.74; 95% CI 0.67 to 0.81; p<0,0001.). Substantial heterogeneity (I^2^ = 84.7%) was detected as shown in the forest plots (*Figure* *2*). The OR predictive interval was 0.48 to 1.13, meaning that no effect or an adverse effect of early surgery might be a plausible finding in a new study. The Mantel-Haenszel OR was 0.79 (95% CI 0.77 to 0.81; p<0.0001), suggesting a modest impact of small studies on the random effects estimate towards more beneficial values.

Bayesian meta-analysis yielded an OR of 0.72 and a 95% credible interval (0.61 to 0.84) the upper bound of which was relatively insensitive to assumptions about prior distribution for random effects. The likelihood of early surgery being found to be beneficial varied between 78% and 82% according to assumptions on priors, suggesting that no or an adverse effect of early surgery could be predicted in about 20% of future studies.

The benefit was resistant to conservative interpretation with sceptical credibility ceilings: only when we considered that there was no chance of any single study convincing us more than about 22% that the effect of early surgery was beneficial did the pooled estimate predict that early and delayed surgery were equivalent.

### Meta-regression analyses

#### Risk of bias

Fourteen studies, seven of low quality [32], [34], [35], [36], [44], [46], [57] and eight of high quality [37], [40], [43], [45], [50], [53], [54], reported prospective adjusted measures of effect. The overall random effects meta-analysis of ORs yielded a pooled estimate favouring early surgery (OR: 0.69; 95% CI: 0.57–0.83; I^2^ = 75.4%). The test for differences between study quality subgroups was not statistically significant (random effects meta-regression P = 0.42). The results are reported in *Figure* *3*.

#### Other confounders

*Table* *3* summarizes meta-regression and *Figure* *4* subgroup results on unadjusted data. In all studies the reporting of mortality stratified for comorbidity was incomplete and we were therefore unable to do a reliable meta-regression analysis of this predictor. None of the covariates studied with meta-regression or subgroup analyses yielded any significant effects on mortality (p≥0.05). However, the power of these analyses is typically low and exploration of confounders may be possibly subject to aggregation bias.

The association between the OR for being operated earlier or later and study baseline risk approached statistical significance, but did not cross it (regression coefficient: −0.14; 95% Bayesian credible interval from −0.40 to 0.09) i.e., the decrease in mortality baseline risk corresponds to a decrease in the difference between early or late surgery (OR close to 1). The probability of the regression coefficient being different from nil was 0.88%.

### Time to surgery and secondary outcomes

We were able to do additional meta-analyses for pressure sores (six studies, 4590 patients, random effects pooled OR 0.48, 95% CI, 0.38–0.60; I^2^ = 0%). The studies were extremely heterogeneous in terms of post-operative complications (i.e. different complications were grouped) and hospital length of stay. Considering the subsample of studies published since 2000, based on an arbitrary period focused on the last decade, mean length of stay varied between seven and 46 days, raising concern that studies differed in their postoperative pathways and hospital discharge policies. These health service differences prevented any meta-analysis of these outcomes. We found data from one study each for post-operative chronic pain and readmission.

### Small study effects

Visual inspection of the contour-enhanced funnel plot (*Figure* *5*) indicated that pooled data did not appear to be heavily influenced by publication bias. This means that slight asymmetry of the plot is possible, with relatively few studies existing midway in the area of non-significance. It is also possible that others are ‘missing’ from this area. Nevertheless Harbord’s test was not statistically significant (P = 0.173).

## Discussion

This meta-analysis of 35 studies showed that elderly patients operated for hip fracture sooner – i.e. within one or two days from hospital admission – have significantly less mortality than patients scheduled for surgery after the second day. After adjustment for age, female prevalence, location, and year, or after omitting low-quality and retrospective studies, this association remained consistently significant. This result was resistant to conservative approaches that increase the confidence attributed to significant effects based on nominal statistical significance. Observational study designs have limitations, but our results do indicate that there probably are differences in mortality outcomes between early and delayed hip fracture surgery, and may even be large in terms of patient benefits. Given the wide diversity of these studies and the frequent lack of control for clinically relevant confounders such as comorbidities, the quantitative results must be seen as merely strongly suggestive, not conclusive.

We observed substantial intra-study heterogeneity that was not explained by any of the study-level variables. Given the absolute majority of non-randomized studies and the variability in settings, health status and comorbidities, adjusting factors, and databases used, some heterogeneity is to be expected. Relying on quantitative inferences generated by observational studies, however, can lead to misleading claims when the overall data suffers from substantial heterogeneity that remains unexplained. On the other hand we explored and found no major differences between studies with adjustments for covariates or unadjusted studies and between studies using clinical or administrative data sources: This might limit the effects of confounders.

In patients immobilized for hip fractures, mortality is influenced by multiple risk factors. Less than half the studies considered comorbidity. Other covariates, such as the centre in multi-centre studies or cognitive impairment, were often ignored. Inadequacies in adjusting for important confounders is an important threat to this systematic review and it is very hard to make inferences or probe their exact depth *ex post*. This might reflect the excessive use of administrative databases rather than medical records to obtain a critical mass of data. Administrative databases are clearly a poor source of information on even major confounders and the rudimentary quality of this type of data has been questioned [25]. Another caveat is that large-scale studies based on administrative databases may have far more weight in a meta-analysis than smaller studies based on meticulous examination of full health records, even using methods to down-weight the spurious precision (i.e. random effects model).

Other threats to internal validity are plausible and relevant to this systematic review. Selection bias is the confounding of treatment effects with population differences: clinicians may without hesitation refer healthier patients for surgery, so differences in mortality tend to be confounded by the indication to surgery. This bias could be combined with another threat: in some studies patients were considered ineligible on the basis of acute medical conditions and surgical delays due to admission and pre-operative management. This builds in the plausible likelihood of group differences that can masquerade as treatment effect. If treatment improves the outcome and patients operated earlier are on average healthier, they could gain more from the treatment, returning to health faster than the controls, thus boosting the differences between groups.

There are other systematic reviews published in a short time on the relation between mortality and hip surgery [59], [60]. Our results confirm the previous findings by Simunovic et al [15]. Although the large number of patients and studies increases the power of our meta-analysis, the precision of effect sizes should not be overinterpreted. Our meta-analysis reports the prediction interval that addresses the actual dispersion of effect sizes across studies and shows little change with more studies. The Bayesian meta-analysis predicts values of OR larger than 1 in about 20% of future studies. Thus, the mean benefit is unlikely to be found across all patients and clinical settings. Early surgery may not save lives, or can even cause more deaths. The interplay of clinical and organizational determinants of quality of care and the potential selection of patients at higher or lower risk of mortality at each hospital makes surgery and admission for hip fracture a complex procedure. In *Table* *4* methods and results of the review by Simunovic are compared to this review to highlight the similar conclusions made by two independent groups.

Many different cut-off times have been used to distinguish early and late surgery: most studies used 24 and 48 hours while a few others used shorter (up to 6 hours) or longer (up to 72 hours) times. Although we grouped studies based on the cut-off time selected, it is difficult to draw a precise line between early and delayed surgery, and it is clear that all these observational comparison groups could still be unbalanced and give rise to significant biases. The optimal timing might be between 12 and 48 hours, identifying two time windows: an immediate timing strategy so that surgery is scheduled on the same or next calendar day after hospital admission and a rapid early timing strategy so that surgery is scheduled within two days of admission.

Although a randomized trial in this context has been opposed because it could be unethical [7], a stronger design such as a randomized trial evaluating the timing of surgery (immediate or rapid) is an attractive idea to reduce the threats supporting causal inferences. This approach is generally accepted in cardiology where there are several examples of randomized trials evaluating the optimal timing of invasive interventions [61], [62]. In our systematic review we found only one RCT examining the effect of delayed hip surgery in the context of a multifaceted intervention [57]. Although this trial suffered by several shortcomings including poor intervention integrity, it can still be considered a key-milestone study because it offered patients a potential genuine equipoise. If observational designs are preferred, we invite investigators to use more quasi-experimental design elements: propensity scores based on medical records have been successfully used in some studies [41], [45] and merit more attention.

In Italy, only one third of patients are operated within three days after admission (region ranges from 11% to 60%) [63]. The feasibility of operating sooner after the injury depends on the efficient use of hospital resources: key contextual elements are the number of surgeons, anaesthetists and operating rooms available and the absence of prolonged clinical assessments due to administrative delays or low-clinical-value investigations. The results of this study encourage hospitals to shorten the time from admission to hip fracture surgery: operating rooms available by night and day 33, 48, application of risk scores at admission [37], multi-disciplinary management 51, and timing as a quality indicator [56], have all been proposed to improve quality. Clearly, further economic analyses are needed to assess the cost-effectiveness of these strategies in various health care systems.

Early hip fracture surgery does appear to provide a survival benefit in comparison with later intervention; it was also associated with a significant reduction in pressure sores. Conservative timing strategies should be limited to patients who will benefit most (i.e. those requiring stabilization) because, besides consuming considerable resources, and physician and nursing time, they may severely affect a patient’s health. Cardiac or renal failure is a compelling reason for delay: cardiologists or nephrologists are consulted to set the timing for surgery, and patients often require additional treatments and tests that take time. This unavoidable delay keeps the patient in bed, increasing the risk of pulmonary, skin and urinary infections and may erode the benefit brought by the specialist approach. Whenever possible the consultation should be completed in 24–48 hours. Administrative delays are unjustifiable. This strategy should be pursued in high- and low-volume hip fracture centres. The early surgery strategy is not intended as a race against time to operate patients in a few hours but everything possible should be done to ensure the majority of patients are operated within one to two days.

## Supporting Information

Table S1 **Electronic search strategies.**
(DOC)
