# Supplementary material for: Timing Matters in Hip Fracture Surgery: Patients Operated within 48 Hours Have Better Outcomes. A Meta-Analysis and Meta-Regression of over 190,000 Patients
Source: PLoS One. 2012 Oct 3;7(10):e46175. doi: 10.1371/journal.pone.0046175 (PMC3463569; doi:10.1371/journal.pone.0046175)
Supplement: Table S1 — Electronic search strategies. (DOC) [file pone.0046175.s001.doc]

*Table S1. Electronic search strategies.*

| 1 | (Hip Fractures"[Mesh]) |
| --- | --- |
| 2 | (hip* OR femur* OR femoral* OR trochant* OR pertrochant* OR intertrochant* OR subtrochant* OR intracapsular* OR extracapsular*) |
| 3 | (fracture*) |
| 4 | 2 AND 3 |
| 5 | 1 OR 4 |
| 6 | (pin* OR nail* OR screw* OR plate* OR arthroplast* OR fix* OR prosthes*) |
| 7 | ("Bone Screws"[Mesh]) |
| 8 | ("Internal Fixators"[Mesh]) |
| 9 | ("Fracture Fixation, Internal"[Mesh]) |
| 10 | ("Bone Plates"[Mesh]) |
| 11 | ("Bone Nails"[Mesh]) |
| 12 | ("Arthroplasty"[Mesh]) |
| 13 | ("Internal Fixator" OR "internal fixators") |
| 14 | (Bone AND (plate* OR nail* OR screw*) |
| 15 | (Arthroplast*) |
| 16 | 6 OR 7 OR 8 OR 9 OR 10 OR 11 OR 12 OR 13 OR 14 OR 15 |
| 17 | ((late OR delay* OR early) AND surgery) |
| 18 | ("pre operative" OR preoperative) |
| 19 | ("timing surgery") |
| 20 | 17 OR 18 OR 19 |
| 21 | ("Hip Fractures"[Mesh]) |
| 22 | ((hip* OR femur* OR femoral* OR trochant* OR pertrochant* OR intertrochant* OR subtrochant* OR intracapsular* OR extracapsular*) AND fracture*) |
| *23* | *21 OR 22* |
| 24 | (pin* OR nail* OR screw* OR plate* OR arthroplast* OR fix* OR prosthes*) |
| 25 | ("Bone Screws"[Mesh]) |
| 26 | ("Internal Fixators"[Mesh]) |
| 27 | ("Fracture Fixation, Internal"[Mesh]) |
| 28 | ("Bone Plates"[Mesh]) |
| 29 | ("Bone Nails"[Mesh]) |
| 30 | ("Arthroplasty"[Mesh]) |
| 31 | ("Internal Fixator" OR "internal fixators") |
| 32 | (Bone AND (plate* OR nail* OR screw*)) |
| 33 | (Arthroplast*) |
| 34 | 24 OR 25 OR 26 OR 27 OR 28 OR 29 OR 30 OR 31 OR 32 OR 33 |
| 35 | ((late OR delay* OR early) AND surgery) |
| 36 | ("pre operative" OR preoperative) |
| 37 | ("timing surgery") |
| 38 | 35 OR 36 OR 37 |
| 39 | 5 AND 16 AND 20 AND 23 AND 34 AND 38 |
